# Atropine sulfate may be effective to recover the unstable hemodynamics in coronary artery spasms related to atrial fibrillation ablation procedures

**DOI:** 10.1002/joa3.13090

**Published:** 2024-06-14

**Authors:** Shunsuke Kawai, Arihide Okahara, Masaki Tokutome, Hirohide Matsuura, Yasushi Mukai

**Affiliations:** ^1^ Department of Cardiovascular Medicine Japanese Red Cross Fukuoka Hospital Fukuoka Japan

**Keywords:** atrial fibrillation, atropine sulfate, catheter ablation, coronary artery spasms, parasympathetic nerve complexes

## Abstract

Coronary artery spasms related to atrial fibrillation ablation procedures could cause lethal ventricular fibrillation or cardiopulmonary arrest. It may be useful to try intravenous atropine sulfate while preparing urgent coronary artery angiography in hemodynamically unstable coronary artery spasms cases to prevent development of the lethal arrhythmias.

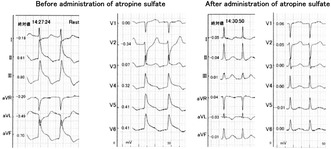

Coronary artery spasms (CASs) related to atrial fibrillation (AF) ablation procedures could cause lethal ventricular fibrillation (VF) or cardiopulmonary arrest (CPA).^1^, ^2^ Although intravenous or intracoronary injection of nitroglycerin is recommended, nitrate administration is sometimes difficult due to the hemodynamics instability. Here, we report two cases in which atropine sulfate relieved CASs and ameliorated hemodynamics.

Case 1 is a 62‐year‐old woman with symptomatic paroxysmal AF. She was referred to our institution for catheter ablation and underwent pulmonary vein isolation with the Heart Light X3 (CardioFocus, Marlborough, MA, USA) endoscopic laser ablation system. ST elevation in the inferior leads occurred 4 min after the insertion of Heart Light Deflectable sheath™ into the left atrium before the first laser ablation to the left superior pulmonary vein was applied (Figure 1A). Sinus bradycardia (HR 44 bpm) and hypotension (systolic BP 60 mmHg) subsequently came up. An intravenous administration of atropine sulfate (1 mg) immediately ameliorated hemodynamics, and the ECG changes were restored (Figure 1B). An urgent coronary artery angiography was performed where neither spasm nor any significant organic stenosis were detected. Thereafter, pulmonary vein isolation using the Heart Light X3 could be accomplished without recurrent hemodynamic instability or ECG changes. She was discharged 2 days after the catheter ablation without vasodilator drugs. AF did not recur during 12 months of follow‐up with no recurrent anginal attack either.

**FIGURE 1:**
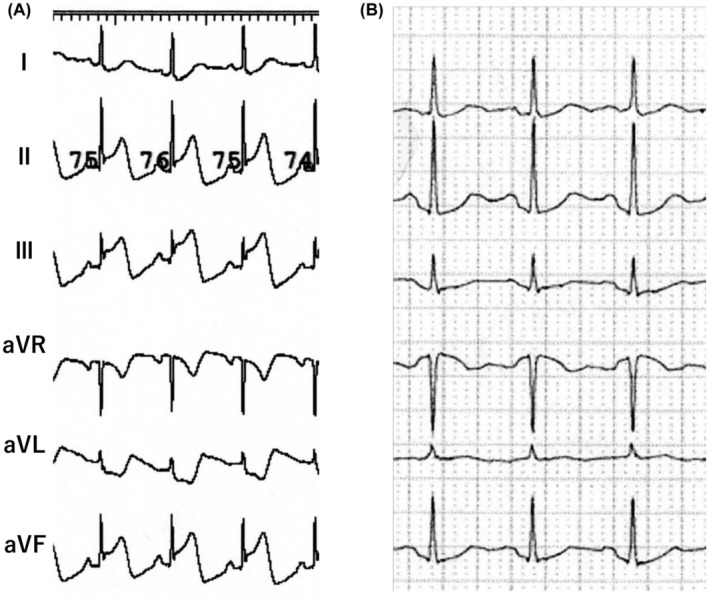
(A) Remarkable ST‐T elevation in the inferior leads and mirror image ST‐T depression in the lateral leads occurred. (B) Intravenous administration of atropine sulfate immediately restored ST‐T elevation in the inferior leads.

Case 2 is a 68‐year‐old man with symptomatic paroxysmal AF. He was referred to our institution for catheter ablation, then underwent pulmonary vein isolation plus left atrial roof ablation with a 28‐mm fourth‐generation cryoballoon (Arctic Front Advance PRO, Medtronic, Minneapolis, MN, USA) 9 months before. He underwent 2nd session catheter ablation for recurrent paroxysmal AF. In addition to right superior pulmonary vein re‐isolation, cavo‐tricuspid isthmus linear ablation was performed with a radiofrequency ablation (TactiFlex SE, Abbott, Chicago, IL, USA) under a stable hemodynamic state. At the end of the session, ST elevation in the inferior/precordial leads occurred immediately after the Swartz™ SL1™ sheaths were removed from the left atrium (Figure 2A), followed by a remarkable hypotension (BP 59/48 mmHg). An intravenous administration of atropine sulfate (1 mg) ameliorated hemodynamics and the ECG changes were restored (Figure 2B). An urgent coronary artery angiography revealed a diffuse spastic response of the both coronary arteries (Figure 3A). Intracoronary nitrate administration relieved the spasm (Figure 3B). He was discharged 3 days after the ablation without vasodilator drugs. AF did not recur during 12 months of follow‐up with no recurrent anginal attack either.

**FIGURE 2:**
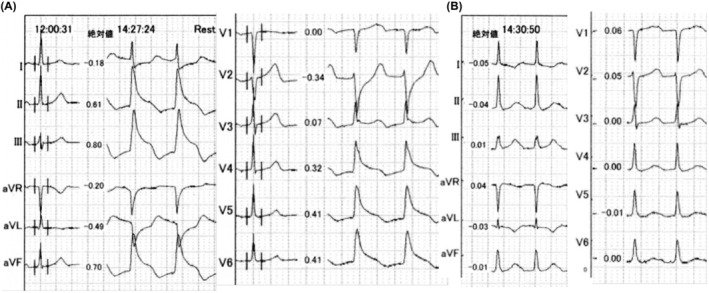
(A) Remarkable ST‐T elevation in the inferior/precordial leads and mirror image ST‐T depression in the lateral leads occurred. (B) ECG changes were restored immediately after intravenous administration of atropine sulfate.

**FIGURE 3:**
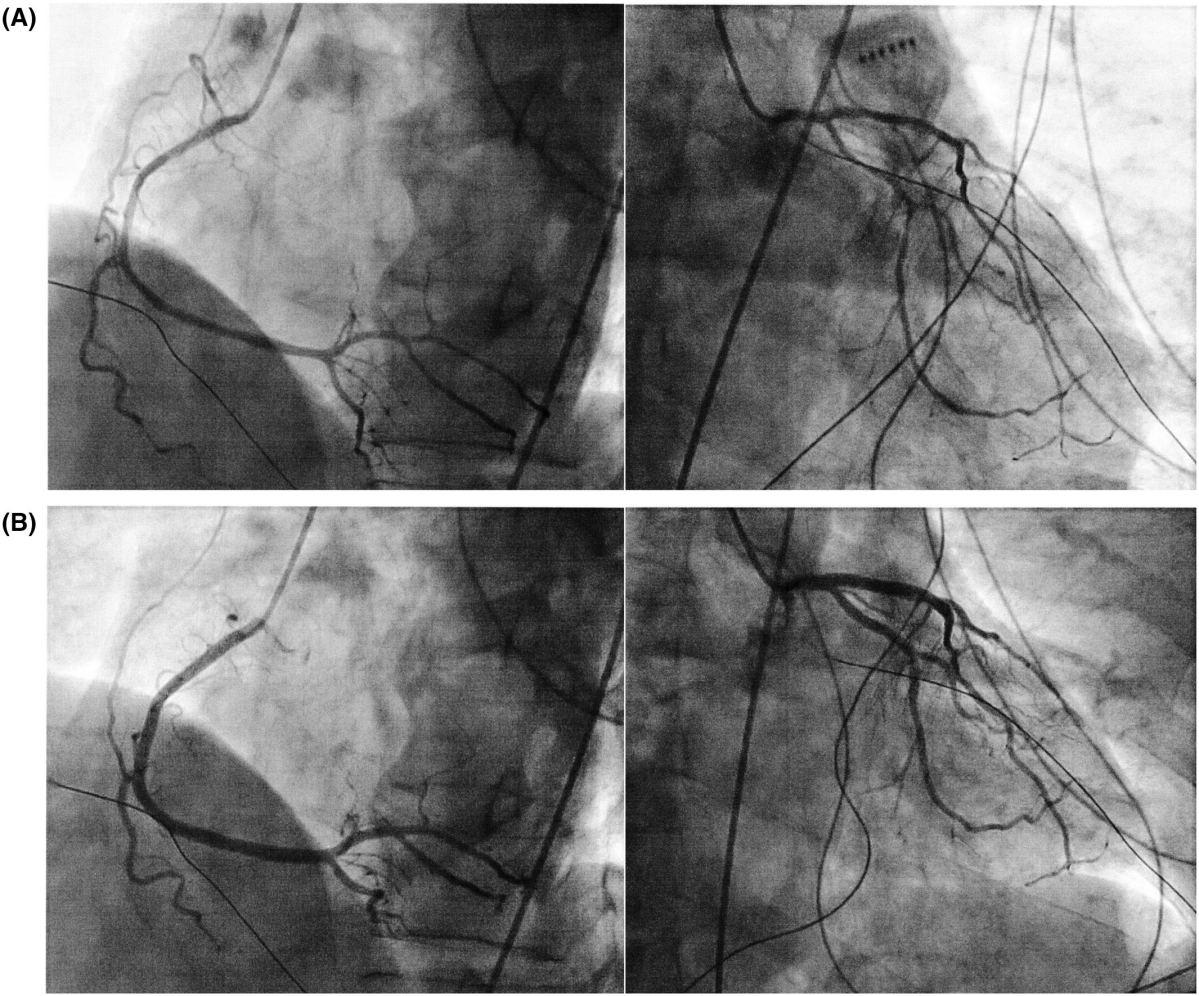
(A) Coronary angiography revealed diffuse spastic response of the both coronary arteries. (B) Intracoronary nitrate administration relieved the spastic state.

The prevalence rate of CASs associated with AF ablation is reported to be 0.19%–0.31%.^3^, ^4^ Although CASs most often occur during the delivery of ablation energy, CASs also occur before/after the ablation procedure.^3^ While ST elevation resolution was obtained spontaneously or quickly after the intravenous administration of nitrate in some cases, Nakamura and colleagues reported CASs developed VF and/or CPA requiring cardiopulmonary resuscitation in seven of 42 patients (17%).^3^

Presumed mechanisms of CASs associated with AF ablation have been reported as follows; direct thermal/cooling effects on the adjacent coronary artery, autonomic nervous system imbalance caused by the affected ganglionated plexus through thermal/cooling injury, stimulation to the high‐density parasympathetic nerve complexes of the atrial septum during transseptal puncture or removal of left atrial sheath^5^ and dexmedetomidine hydrochloride, an α‐2 adrenergic receptor stimulant, mediating vasoconstriction.

In a strict sense, it may be possible that transient air embolism caused transient ST elevation because coronary angiographies were performed after ST elevation resolution was obtained both in our cases. Air emboli can be introduced from the transseptal sheaths and migrate into the right coronary artery because the right coronary cusp is positioned at the superior aspect of the heart when the patients are in the supine position. However, in our two cases, CASs occurred during the transseptal puncture procedure or just after the removal of left atrial sheaths, both of which could stimulate the high‐density parasympathetic nerve complexes of the atrial septum. It may be true that recovery from the parasympathetic reflex by atropine sulfate increased the coronary flows, then strong CASs recovered as a result. From these clinical experiences of CASs related to AF ablation, we conclude that it may be useful to try intravenous atropine sulfate while preparing urgent coronary artery angiography in hemodynamically unstable CASs cases to prevent development of the lethal arrhythmias.

## CONFLICT OF INTEREST STATEMENT

The authors declare no conflict of interests for this article.

## PATIENT CONSENT STATEMENT

Written informed consent was obtained from the patients to publish this report.
